# Exploration of the relevance and comprehensibility of the European Organization for the Research and Treatment of Cancer Sexual Health Questionnaire among Danish young adults aged 18–39: a national cross-sectional study

**DOI:** 10.1186/s41687-025-00988-w

**Published:** 2025-12-31

**Authors:** Maria Aagesen, Helle Pappot, Karin Piil, Ligita Paskeviciute Frøding, Emma Balch Steen-Olsen, Elfriede Greimel, Line Bentsen

**Affiliations:** 1 Department of Oncology, Copenhagen University Hospital, Rigshospitalet, Blegdamsvej 9, Copenhagen, 2100 Denmark; 2 Occupational Science, User Perspectives and Community-Based Interventions, Department of Public Health, University of Southern Denmark, Campusvej 55, Odense, 5230 Denmark; 3 Network for Adolescents and Young Adults Cancer Research in Denmark (NAYAcare DK), Danish Comprehensive Cancer Center (DCCC). www.dccc.dk, Aarhus, Denmark; 4 Department of Clinical Medicine, University of Copenhagen, Blegdamsvej 3B, Copenhagen, 2100 Denmark; 5 Department of Gynecology, Copenhagen University Hospital, Rigshospitalet, Copenhagen, 2100 Denmark; 6 Department of Obstetrics and Gynecology, Medical University of Graz, Auenbruggerplatz 14, Graz, 8036 Austria

**Keywords:** Young adults, Oncology, Sexual health, Quality of life

## Abstract

**Background:**

The EORTC Sexual Health Questionnaire (EORTC QLQ-SH22) assesses sexual health-related quality of life in adult cancer patients. However, it may not fully address age-dependent issues in young adults aged 18–39 at diagnosis. This study aimed to explore the questionnaire’s relevance and comprehensibility for young adults with cancer and identify topics needing elaboration or missing.

**Methodology: in:**

this cross-sectional study 60 young adults with cancer across treatment stages rated item relevance on a Likert scale and gave qualitative feedback through a “think-aloud” process and semi-structured interviews. Quantitative analyses examined item relevance using mean scores and predefined thresholds. Qualitative data were analysed using reflexive thematic analysis.

**Results:**

Twenty-one of the 22 items were rated relevant. Participants suggested refining wording to improve comprehensibility. Interviews indicated a need to expand coverage of intimacy, communication with professionals, body changes and body image, and partnership. Missing topics included singlehood, cancer before sexual debut, contraception and fertility, and gender diversity.

**Conclusions:**

The EORTC QLQ-SH22 is relevant for young adults with cancer but may be improved through clearer wording, elaboration of key topics, and inclusion of missing items. An international content validity study in this population is recommended to guide future development.

**Supplementary Information:**

The online version contains supplementary material available at 10.1186/s41687-025-00988-w.

## Background

Globally, approximately one million young adults (18–39 years) are diagnosed with cancer each year [1]. During and after treatment, young adults may experience challenges that affect sexual health–related quality of life (HR-QoL), which refers to the aspects of overall health-related QoL encompassing physical, emotional, mental, and social well-being in relation to sexuality [2–6]. Sexual health itself includes sexual orientation, gender identity, sexual expression, relationships, pleasure, sexual function, and pregnancy and abortion choices [2].

Unlike their older adults counterparts, who more often are more sexual experienced, have established relationships and completed their reproductive years, young adults are at a pivotal life stage where exploring sexual identity, initiating long-term relationships, and considering family planning are central concerns [7–9]. The interplay of these life-stage factors with the physical and psychological side- and late effects of cancer and its treatment, creates distinct sexual health needs for young adults [6, 10–14].

Cancer treatments such as chemotherapy may lower hormone levels that influence sexual desire, while in men, surgery or radiotherapy to the genital region can damage nerves or blood vessels, potentially causing erectile difficulties. In women, similar treatments—along with anti-hormonal therapy—can lead to vaginal dryness and atrophy [15]. Furthermore treatment-related fatigue, changes in body weight, surgical scars, and altered body image may also reduce sexual interest or satisfaction in both sexes [15–17]. Many of these effects can persist long after treatment, becoming late effects with risk of reducing sexual HR-QoL [15].

Young adults with cancer often wish to discuss sexual health concerns with healthcare professionals (HCPs) [18, 19]. However, these conversations rarely take place [20], as many HCPs perceive sexual health as a taboo topic [21]. When discussions do occur, they are often limited to medical aspects rather than broader sexual health concerns [22]. Additionally, young adults may struggle to bring up these issues due to their sensitive nature [10]. As a result, sexual health is often inadequately addressed, despite recommendations in multiple guidelines [19, 23–25], leading to unmet needs in this patient group.

To facilitate discussions on sexual health, young adults suggest using predefined topics and dialogue tools to ensure all relevant issues are addressed [19]. Several validated patient-reported outcomes measures (PROMs) exist that evaluate various aspects of sexual health independently, such as instruments measuring sexual dysfunction or body image [26]. However, these PROMs are limited in their ability to capture all relevant domains and dimensions of sexual health. To address this gap, the self-reported PROM European Organization for Research and Treatment of Cancer Sexual Health Questionnaire (EORTC QLQ-SH22) was developed to systematically identify and address sexual health concerns in relation to QoL [27, 28]. The initial item generation followed the EORTC Quality of Life Group’s guidelines for developing questionnaires, involving a comprehensive group of stakeholders, including patients with different cancer diagnoses, healthcare professionals, and subject matter experts. This multi-perspective approach ensured that items reflected both clinically relevant issues and patient-prioritized concerns [27]. Previous psychometric validation study has provided evidence for the EORTC QLQ-SH22 questionnaire’s reliability and the content and construct validity in mixed adult cancer populations. However, in the validation study only 5% (23 out of 444 participants) were aged 20–35 years, making them the youngest group in the study [29]. This limited representation of young adults may affect the questionnaire’s relevance and validity for this patient group, particularly in terms of content and face validity. Since questionnaire items should reflect patients’ perspectives to ensure they measure concepts relevant to the target group [27, 29, 30], the EORTC Quality of Life Group recommends further validation of the EORTC QLQ-SH22 in young adults with cancer [27, 29]. As an initial step in insuring that the EORTC QLQ-SH22 is relevant to capture sexual HR-QoL in young adults, this initial exploratory study may inform whether future validation efforts and questionnaire refinement is needed.

## Methods

The aim of this study is: (1) to explore the relevance and comprehensibility of EORTC QLQ-SH22 for young adults with cancer, and (2) to identify any topics needing further elaboration or missing in the questionnaire.

### Design

This is a cross-sectional study exploring the relevance and comprehensibility of the EORTC QLQ-SH22 questionnaire combining quantitative and qualitative approaches, inspired by the EORTC Quality of Life Group guidelines for developing questionnaires [27, 31].

### Setting and recruitment

In this study “young adults” were defined as individuals aged 18–39, consistent with the definition commonly used in Denmark and other comparable European countries for cancer survivorship serviced and research [15]. The eligibility criteria therefore included a confirmed cancer diagnosis at any tumor site and stage, age 18–39 at diagnosis, the ability to understand Danish, and no cognitive impairment. Inclusion of all tumor sites and stages ensured broad representation of the young adult cancer experience. Danish language proficiency was required to accurately assess comprehensibility, and individuals with cognitive impairment were excluded to ensure reliable and meaningful responses.

Participants were recruited from April 2023 throughout August 2024 by the clinical staff at oncological or hematological units across Denmark where young adults received treatment and/or follow-up care for a confirmed cancer diagnosis. Recruitment was also conducted through closed Facebook groups for young adults with cancer administered by HCPs at the hematological and oncological departments in Denmark and the social media channel of the national patient organization for young people with cancer (Ung Kræft) administered by the Danish Cancer Society [32]. For participants recruited via social media, the cancer diagnosis was verified using a structured liability questionnaire that included details on diagnosis date, treatment received, and treatment location. Responses were cross-checked for consistency, as the research team did not have access to medical records. Purposive sampling was employed to ensure variation in the population. Efforts were made to recruit an equal number of men and women, a broad range of tumor sites, and an equal distribution of participants across the following categories: (1) young adults treated with surgery only without any adjuvant treatment, (2) young adults in first-line curative intended oncological treatment with or without surgery, (3) young adults in life-extending oncological treatment with or without surgery, and (4) young adults in follow-up after first-line therapy with or without surgery with no evidence of disease, spanning six months to five years post-treatment. Participants could only be classified in one of the four categories. This was based on the recruitment matrix from the EORTC QLQ-SH22 psychometric validation study [29]. No participants declined to participate during the recruitment process.

### Questionnaire

The EORTC QLQ-SH22 consists of 22 questions (items) assessing various domains related to sexual health, including sexual interest, activity, satisfaction, and distress [33]. Additionally, it addresses body image and intimacy issues [34]. The questionnaire’s structure enables the identification of specific sexual health issues that may require targeted interventions, thereby facilitating person-centred care.

Each item in the questionnaire is rated on a Likert scale, rated with “not at all"=1, “a little”=2, “quite a bit”=3 and “very much"=4, allowing for a nuanced evaluation of the patient’s experiences and concerns [33]. This level of detail captures the multifaceted impacts of cancer and its treatment on sexual health, providing clinicians and researchers with valuable insights into the sexual HR-QoL among people diagnosed with cancer [27, 29].

### Data collection

Data collection involved individual face-to-face, or telephone interviews to collect quantitative and qualitative data. The interviews were conducted by three HCPs (M.A., E.S.T., and L.B.) who are all experienced in conducting semi-structured interviews. The interviewers were not otherwise involved in the participants’ cancer trajectory.

Each interview began with demographic and cancer-related information (presented in Table 1). This was followed by a structured three-step process:

**Table 1:** Demographic and cancer-related characteristics (*n* = 60)

	*N* (%)
*Gender*	
Female	24 (40)
Male	36 (60)
*Age groups*	
18–29	35 (58)
30–39	25 (42)
*Relationship status*	
Partner	35 (58)
Single	25 (42)
*Living situation*	
Living with a partner/family	23 (38)
Living with parents	10 (17)
Living with others	8 (13)
Living alone	19 (32)
*Education*	
Compulsory school or less	29 (48)
Post compulsory school education	17 (28)
University level	14 (23)
*Employment status*	
Full time job or student	29 (49)
Part time job or student	9 (15)
Sick leave	14 (23)
Other^a^	8 (13)
*Tumour site*	
Breast	10 (17)
Gynecological cancer	3 (5)
Testes	19 (32)
Central nervous system	5 (8)
Leukemia	6 (10)
Lymphoma	12 (20)
Other^b^	3 (5)
*Time since diagnosis*	
Under 1 years	27 (45)
1–2 years	13 (22)
Over 2 years	20 (33)
*Treatment received* ^*c*^	
Chemotherapy	38 (63)
Radiation	14 (23)
Surgery	40 (67)
Hormonal	3 (5)
Immunotherapy	7 (12)
Bone marrow transplant	1 (2)
*Cancer trajectory categories*	
Participants treated with surgery only without any adjuvant treatment (Category 1)	16 (27)
Participants in first-line curative intended oncological treatment with or without surgery (Category 2)	13 (22)
Participants in life-extending oncological treatment with or without surgery (Category 3)	5 (8)
Participants in follow-up after first-line therapy with or without surgery with no evidence of disease, spanning six months to five years post-treatment (Category 4)	26 (43)
*Status of disease*	
Currently on curative intended treatment	10 (17)
Life-extending oncological treatment	5 (8)
Follow-up	45 (75)
*ECOG*,* performance status*	
Fully active	27 (45)
Restricted	27 (45)
Self-care possible	6 (10)
*Comorbidity*	
Yes	13 (22)
No	47 (78)

^a^Maternity leave, unemployed or flexible job situation, ^b^Head and neck, lung cancer, sarcoma, multiple solid tumors, polycythemia vera, ^c^Multiple answers were possible

ECOG = Eastern Cooperative Oncology Group

1. Quantitative data collection regarding relevance: Item relevancy was defined as to what extent an item directly reflected sexual health issues [35]. Participants reviewed each item and rated its relevance in capturing their sexual health concerns on a 4-point Likert scale (1 = not at all, 2 = a little, 3 = quite a bit, 4 = very much).
2. Qualitative data collection regarding cognitive feedback: Participants were asked to “think aloud,” providing real-time feedback on item wording and comprehension.
3. Qualitative data collection regarding the overall experiences of the questionnaire: Participants engaged in a semi-structured interview based on an interview guide including questions about whether any topics or issues relevant to their sexual health needed elaboration or were missing in the questionnaire.

### Data analysis

First, the relevance of each item was explored by calculating the proportion of participants who found it relevant, presented as number and percentage. The Likert scale ratings for each question were statistically analyzed to determine the mean score and standard deviation. A question was deemed relevant if it met the following thresholds: (1) a mean score > 1.5, (2) a prevalence of Likert scores 3 or 4 exceeding 50%, and (3) fewer than 10% missing responses [27].

Second, all items eliciting comments during the “think loud” process were examined to identify wording and comprehension issues, along with potential suggestions for improving clarity.

Third, the semi-structured interviews were analyzed using a combination of Malterud’s systematic text condensation [36] and Braun and Clark’s reflexive thematic analysis [37, 38]. M.A. and L.B. independently familiarized themselves with the data consecutively. They inductively coded details from each interview, identifying themes related to the young adults’ thoughts on sexual health within the questionnaire and their suggestions for enhancing the questionnaire’s relevance to the population. The two authors collaborated iteratively to categorize similar themes and resolve any disagreements through discussions until consensus was reached.

Data saturation was achieved during the data collection process, as no new themes or insights emerged in the later interviews. This ensured the adequacy and completeness of the data for our analysis.

## Results

Sixty young adults participated in the study. The participants had a mean age of 29 years at diagnosis (range 18–39 years). Of the group, 24 participants were females (40%) and 25 were single (42%). Sixteen participants (27%) were treated with surgery only (Category 1), 13 participants (22%) were in first-line curative intended treatment with or without surgery (Category 2), 5 participants (8%) were in life-extending treatment (Category 3) and 26 participants (43%) were un follow-up (Category 4). The cancer types represented leukemia, lymphoma, central nervous system cancer, breast cancer, gynecological cancer, and testicular cancer. All sociodemographic and cancer related characteristics are presented in Table 1.

Forty-two interviews were conducted electronically (online or via telephone) and 18 were conducted face-to-face in a quiet office at the Copenhagen University Hospital – Rigshospitalet. The interviews had an average duration of 27.5 min, ranging from 14 to 55 min.

### Relevance of each question

All items achieved a mean score above 1.5 (see Table 2). The lowest mean scores were observed for Item 5 (Mean = 2.38, SD = 1.29), Item 1 (Mean = 2.78, SD = 1.10), and Item 21 (Mean = 2.88, SD = 1.02) (see Supplementary File 1). Notably, Item 5 was the only item with less than 50% rated with a Likert score of 3 (= Quite a bit relevant) or 4 (= Very much relevant) (47%). Ratings for all items spanned more than two points. While missing responses were identified in two of the 22 items, none exceeded 10% for any item (see Table 2). Therefore, 21 of the 22 items were deemed relevant by the participants.

**Table 2:** Evaluation of item relevance

During the last 4 weeks:	Mean score (SD)	Scoring 3 or 4, %	Missing data, *N* (%)
1 How important to you is an active sex life?	3.43 (0.76)	90	0 (0)
2 Have you had decreased libido?	3.50 (0.79)	92	0 (0)
3 Have you been satisfied with your level of sexual desire?	3.05 (0.94)	75	0 (0)
4 Have you been satisfied with your sex life?	3.02 (1.04)	67	0 (0)
5 Have you been worried about being incontinent (urine/stool)?	2.38 (1.29)	47	0 (0)
6 Has fatigue or a lack of energy affected your sex life?	3.63 (0.73)	92	0 (0)
7 Has the treatment affected your sexual activity?	3.40 (0.95)	83	0 (0)
8 Have you been worried that sex would be painful?	3.12 (1.08)	73	0 (0)
9 Have you had communication with health professionals about sexual issues?	3.03 (1.02)	73	0 (0)
10 Have you been satisfied with the communication about sexual issues between yourself and your partner?	3.10 (1.08)	73	0 (0)
11 Have you been worried that your partner may cause you pain during sexual contact?	2.78 (1.10)	60	0 (0)
12 Have you been satisfied with your level of intimacy?	3.12 (0.97)	78	0 (0)
13 Have you felt insecure regarding your ability to satisfy your partner?	3.42 (0.88)	83	0 (0)
14 *For men only*: Were you confident about obtaining and maintaining an erection when you had sex?	3.53 (0.69)	89	0 (0)
15 *For men only*: Have you felt less masculine as a result of your disease or treatment?	3.35 (0.94)	75	1 (3)
16 *For women only*: Have you felt less feminine as a result of your disease or treatment?	3.79 (0.50)	96	0 (0)
17 During the last 4 weeks: Have you been sexually active?	3.27 (0.91)	85	0 (0)
18 If yes in Q17: Has sexual activity been enjoyable for you?	3.41 (0.77)	87	0 (0)
19 If yes in Q17: Have you been satisfied with your ability to reach an orgasm?	3.22 (0.89)	77	0 (0)
20 If yes in Q17: Have you felt pain during/after sexual activity?	3.33 (0.92)	80	0 (0)
21 If yes in Q17: To what extent did you feel sexual enjoyment?	2.88 (1.02)	67	0 (0)
22 *For women only*: Have you experienced a dry vagina during sexual activity?	3.43 (0.82)	83	1 (4)

An item was deemed relevant if it met the following thresholds: (1) a mean score > 1.5, (2) a prevalence of Likert scores 3 or 4 exceeding 50%, and (3) fewer than 10% missing responses [27]

### Wording and comprehension

In total, 125 comments were recorded across all 22 items. In Table 3, the most frequent comments regarding difficulties and confusing items are categorized and summarized. Some participants highlighted that items including phrasing with “masculine” and ”feminine” seemed inappropriate and outdated as gender generally is perceived more fluent nowadays.

**Table 3:** Summarization of frequent comments

Item	Difficulty understanding	Confusing	Suggested wording
1	How is “sexually active” defined?	Does it include sexual activity with a partner and/or oneself?	
3		Determining levels of sexual desire is challenging, making the question difficult to answer.	
5	What does the word “incontinent” mean?		
11		It is difficult to tell the difference between Q11 and Q8.	
12	This is an abstract question. How is intimacy defined?	Determining levels of intimacy is challenging, making the question difficult to answer.	
14	This is an odd phrasing with the word “confident”.		Maybe more a “concern”.
17	It is difficult to quantify sexual activity as from “a little” to “very much”	Why isn’t this question asked first in the questionnaire?	
19 + 21		It is hard to distinguish Q19 and Q21 from Q18.	
Other		Difficult to recall from the last four weeks	
Other		Difficult to distinguish whether the symptoms and issues are attributed cancer or other life circumstances.	
Other	Items including the word “partner”		Consider using “another person” or “partner(s)” to acknowledge that individuals may have more than one partner.

### The participants’ experience of the questionnaire and topics assessed as either sufficient, required elaboration, or missing

The participants discussed those topics including sexual activity, libido, sexual satisfaction, sexual pain, incontinence, erection confidence, and vaginal dryness were sufficiently covered in the questionnaire.

**Fig. 1:**
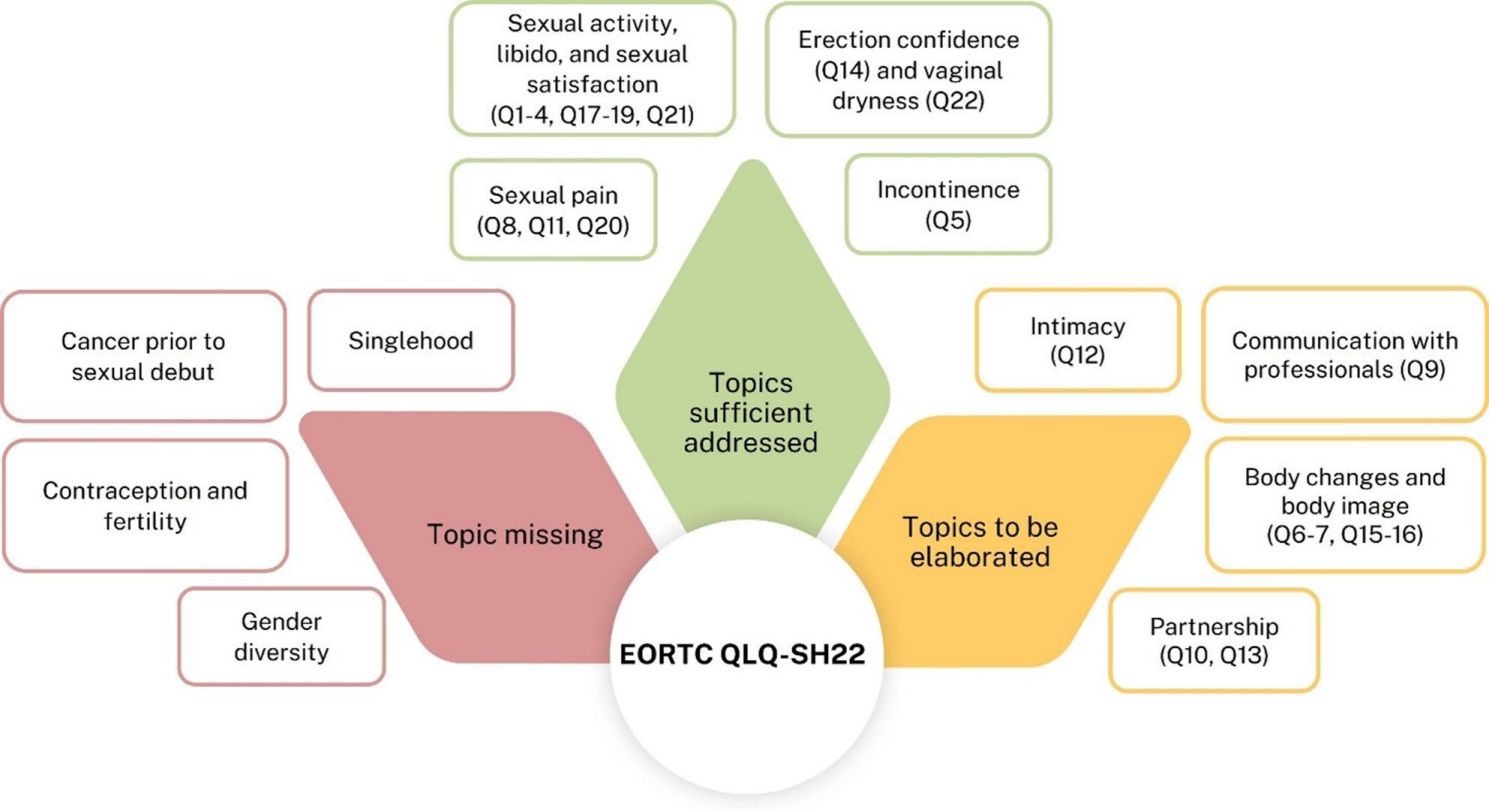
Shows the topics categorized as sufficient, requiring elaboration, or missing, along with the corresponding items from the EORTC QLQ-SH22 questionnaire that address these topics

However, interviews indicated a need to elaborate on topics such as intimacy, communication with professionals, body changes and body image, and partnership. Finally, the participants were missing topics including singlehood, cancer before sexual debut, contraception and fertility, and gender diversity in the questionnaire (Fig. 1).

## Topics needing elaboration in the questionnaire

### Intimacy

Many participants emphasized the need to expand coverage of intimacy, reflecting on the emotional and physical closeness shared with partners beyond sexuality. They highlighted challenges in maintaining intimacy during and after treatment, particularly in preserving connection despite the physical and psychological changes caused by cancer. Moreover, participants expressed a need for support in discussing intimacy with HCPs, advocating for more nuanced conversations addressing emotional connection alongside sexual function. One participant noted:

*Intimacy has become much more important since I became ill*,* so there should be more questions related to this… And something about changes in relationships and closeness before and after the illness. (Interview 37*,* young woman*,* lung cancer)*

This reflects the desire for the questionnaire to capture both relational and emotional dimensions of sexual health, highlighting that addressing intimacy in greater depth could enhance its relevance for young adults navigating relational changes during and after cancer treatment.

### Communication with professionals

Several participants argued the importance of elaborating items about professional guidance on sexual health during and after cancer treatment, raising concerns about the availability and timing of such counselling. Additionally, they highlighted specific needs for information about how side effects from cancer treatments can affect sexual health, such as hormonal changes or physical discomfort. One participant explained:

*More questions about conversation with healthcare professionals about sexual topics would be beneficial (Interview 13*,* young man*,* testicular cancer)*

This highlights the perceived gap in current guidance. Another added the challenges of initiating discussions about sensitive topics:

*Questions about whether it is difficult to reach out to healthcare professionals (Interview 31*,* young woman*,* gynaecological cancer)*

These insights suggest that the questionnaire could be strengthened by capturing not only the availability of professional support but also the perceived accessibility and adequacy of sexual health counselling.

### Body changes and body image

The experience of body changes and its impact on body image during and after cancer treatment was expressed as something that needed to be elaborated more specifically on. Concerns about weight changes, scarring, and hair loss affected their sense of attractiveness and comfort in intimate situations, often leading to decreased confidence, especially with (new) partner. Participants also highlighted the need for items focusing on satisfaction with one’s appearance and addressing body image concerns within the context of sexual health. One participant described these challenges, stating:

*More about how appearance affects sex drive*, e.g.,* weight gain. It has a significant impact on how sexy you feel. Being able to tolerate looking at yourself is crucial for whether you feel like having sex. How do you feel in your own body? (Interview 33*,* young woman*,* breast cancer)*

Another participant emphasized the need to consider broader self-perception and mental well-being.

*Maybe include questions regarding self-perception*,* sexual confidence*,* mental state (Interview 27*,* young woman*,* other cancer diagnoses)*

These accounts indicate that incorporating items addressing body satisfaction, self-perception, and emotional adjustment could provide a more comprehensive understanding of sexual health and intimacy for young adults during and after cancer treatment.

### Partnership

Participants highlighted the importance of addressing how their cancer and its treatment could influence sexual interactions and partner dynamics, including communication about cancer’s sexual implications such as side effects and adjustments to sexual activity. Additionally, they stressed the need for support in navigating these conversations and maintaining openness about their experiences, as well as understanding their partners’ perceptions and reactions to changes in sexuality. For example, one participant reflected:

*It is important to manage expectations and avoid mismatches with your partner. Being able to talk about sex and illness is more important than functionality. (Interview 55*,* young man*,* testicular cancer)*

Another participant pointed out the need to consider the partner’s perspective:

*Something about how sexuality affects my relationship with a partner. The issues around sexuality*,* how does it affect the partner? (Interview 2*,* young woman*,* breast cancer)*

These insights suggest that including items in the questionnaire about communication, mutual expectations, and relational impacts could help capture the broader dimensions of sexual health for young adults with cancer and their partners.

## Topics missing in the questionnaire

### Singlehood

Participants highlighted the need for more attention to the experiences of a single individual, including issues of being single after a cancer diagnosis and navigating its potential impact on future relationships. They highlighted that being single introduces unique challenges, such as navigating intimacy with new partners and discussing potential sexual side effects with future partners. One participant explained:

*When you are single*,* your appearance affects your sexual health*,* and whether you feel like being intimate is greatly influenced*,* especially with “a stranger”. Your appearance and self-image play a role in your sexual health. (Interview 5*,* young woman*,* breast cancer)*

Others noted that the questionnaire seemed primarily oriented toward those in relationships, pointing out the need for items capturing concerns about dating and forming new partnerships after a cancer diagnosis:

*More focus on being single – it seems that the questionnaire is for those in relationships. (Interview 4*,* young woman*,* breast cancer)*

*More focus on the issue of finding a partner after diagnosis (dating) (Interview 17*,* young man*,* central nervous system cancer)*

These reflections indicate that including questions addressing singlehood could better capture the sexual health needs of all young adults with cancer, not only those in established relationships.

### Cancer prior to sexual debut

A few participants noted that the questionnaire lacked items for individuals who had not been sexually active before their cancer diagnosis. They emphasized that concerns related to sexual health as a virgin, such as navigating first sexual experiences, understanding changes in sexual desire, and coping with body change, were insufficiently addressed. One participant illustrated this gap, stating:

*Focus on being diagnosed with cancer as a young adult virgin and thoughts on sex (Interview 59*,* young man*,* testicular cancer)*

This suggests that including items specifically tailored to those who have not yet initiated sexual activity could provide a more inclusive understanding of sexual health needs among young adults with cancer.

### Contraception and fertility

Participants highlighted their fears about the ability to reproduce after cancer, and how these worries could influence sexual activity and intimate relationships. One participant reflected on this connection, noting that:

*Thoughts on sexual health are affected by fertility issues (Interview 5*,* young woman*,* breast cancer)*

Some also pointed out that the questionnaire lacked attention to contraception and the risk of unplanned pregnancy after treatment, with one participant asking:

*Missing questions about contraception*,* fear of pregnancy*,* has it affected sexual activity? (Interview 4*,* young woman*,* breast cancer)*

These reflections suggest that including targeted items on fertility and contraception could better capture the complexities of sexual health experiences among young adults with cancer.

### Gender diversity

Participants emphasized the absence of items addressing LGBTQ + sexual health and suggested including items beyond heteronormative frameworks. Several noted that the current phrasing did not fully capture diverse gender identities or experiences, and that a more inclusive approach was essential for reflecting the realities of young adults with cancer. One participant highlighted that gender identity shapes both emotional and bodily experiences of sexuality:

*Questions about mental issues*,* not just physical ones*, i.e.,* gender identity*,* guilt*,* and body perception (Interview 31*,* young man*,* leukemia)*

Others pointed out that the terminology used in the questionnaire felt outdated, with one remarking that:

*It is very outdated phrasing with masculine and feminine – it is not so divided anymore (Interview 55*,* young man*,* testicular cancer)*

This quote was echoed by another participant who expressed a need for a more flexible and representative framework, stating the following:

*I think the questionnaire should be less gendered and more nuanced (Interview 8*,* young man*,* testicular cancer)*.

Together, these viewpoints illustrate a clear call for inclusive, contemporary, and sensitive language that accommodates the full spectrum of gender identities and expressions.

## Discussion

This study explored the relevance and comprehensibility of the EORTC QLQ-SH22 in young adults with cancer. While 21 of the 22 items were deemed relevant, the participants found certain items unclear or confusing, affecting the overall comprehensibility of the questionnaire. Additionally, participants expressed that some topics required further elaboration, and some topics were missing.

When compared to an older population, the young adults’ mean relevance scores were consistently higher for all items, emphasising the importance of sexual health for this age group [27]. Only one item - about incontinence (Item 5) - was rated “quite a bit” or “very much” relevant by less than 50% of the participants. This finding aligns with studies in older populations on the comprehensibility of the EORTC QLQ-SH22, which similarly note that incontinence may be relevant only for a subset of the cancer population, regardless of age [27, 39].

It was not surprising that several topics were found to require further elaboration or entirely missing as the EORTC QLQ-SH22 was primarily developed and validated in an adult cancer population (20–91 years at diagnosis) [27]. Similar findings have been observed in other questionnaires such as the EORTC QLQ-30 questionnaire, where an adolescent and young adult-specific version recently has been developed to address the unique HR-QoL needs of this age group [40–42]. In addition, the missing topics identified by the participants in our study were not unexpected as they align with themes previously recognized as significant for young individuals navigating a cancer trajectory [15, 19, 43]. Specifically Cherven et al. [6] argues, that young adults with cancer are particularly vulnerable to unmet sexual health needs related to gender diversity, contraception, sexual function, body image, and romantic/sexual relationships.

The importance of addressing intimacy has been found in a previous study, which highlights that for young adults with cancer intimacy becomes increasingly important during a cancer trajectory [44]. However, maintaining intimacy may also be difficult, as sexual activity is often associated with pain during and after cancer treatment, particularly among those with cancer in the reproductive organs [19]. This highlights the relevance of addressing this issue more thoroughly in a sexual health questionnaire for young adults.

Research suggests that cancer and cancer survivorship can be stigmatizing, which may contribute to the challenges young adults with cancer face in dating [45, 46]. Additionally, evidence shows that cancer survivors often encounter difficulties in forming romantic relationships [10], with lower marriage rates among cancer survivors compared to the general population [47, 48]. These challenges can significantly impact the QoL [43, 49, 50]. However, other research indicates that individuals without a history of cancer are open to dating cancer survivors, suggesting that stigma may not always play a major role in this group [51]. Despite these mixed findings, participants in our study emphasized the importance of including this topic in the questionnaire. Notably, the experience of being single and seeking a partner is not unique to young adults with cancer [52], proposing that items addressing singlehood are relevant for individuals across all ages.

Young adults with cancer often find that sexual health is closely intertwined with fertility [19]. Consistent with previous research, our study found that concerns about fertility are significant for many young adults at the time of diagnosis and throughout their cancer trajectory [6, 53–56]. This underscores the potential value of including items about fertility and contraception in the EORTC QLQ-SH22. However, fertility may not be relevant for all young adults with cancer, particularly those who already have the children they desire [57]. Furthermore, it could be argued that fertility-related topics might be better addressed in a dedicated questionnaire, especially as the EORTC Quality of Life Group is currently developing a tool specifically focused on fertility and QoL in cancer patients [58].

Focus on gender diversity was emphasized by the participants in our study as a necessity for creating a more inclusive questionnaire. Similarly, Aubin et al. [26] recommend addressing gender orientation in sexual health consultations with young adults with cancer. Also, Cherven et al. [6], argues that given the sexual and gender fluidity is increasingly dynamic among young adolescents and young adults, sexual health interventions that are centred to the unique needs of sexual minorities are needed. However, research shows that HCPs often struggle to address sexual orientation and gender identity diversity in cancer care [59, 60]. Including items about gender diversity could provide a valuable conversation tool, helping HCPs navigate these discussions with young adults. Additionally, Aubin et al. [26] suggest focusing on whether and how cancer affects a first sexual encounter, a recommendation that aligns with feedback from participants in our study.

Challenges with the conceptualization of key terms in the questionnaire were expressed in this study particularly the terms “sexual activity” and “intimacy”. Difficulties with conceptualizing “sexual activity” were not unexpected as similar challenges were identified in the cross-cultural development study of the EORTC QLQ-SH22 questionnaire, indicating that this challenge is age-independent [27].

Finally, participants also reported difficulties distinguishing whether symptoms or challenges were related to cancer and/or its treatment, other life circumstances, or unrelated factors, raising the question of whether such symptoms should be reported. This challenge has been noted in previous validation studies of EORTC questionnaires [61, 62]. According to Groenvold et al. [62], patients should not focus on the cause of their symptoms but instead report the extent to which the symptoms or challenges occurred within the past four weeks. Although, this premise must be clarified in the questionnaire.

This study indicates that the EORTC QLQ-SH22 questionnaire may not fully capture the sexual health needs of young adults with cancer. Further research should assess its applicability and content validity in an international young adult cancer population to inform any revisions needed to better address this group’s needs. A key strength of this study is the sample size and its diversity of the study population, which includes variation in age, gender, relationship status, educational level, and tumor site. By including patients currently undergoing cancer treatment, along with those in short and long follow-up periods, we enhanced the study’s external validity by representing young adults with cancer across the entire cancer trajectory.

Although purposive sampling ensured variation across sex, tumor sites, and treatment trajectories, it may also have introduced systematic biases. Recruitment through clinical units may have favored inclusion of young adults more affected by sexual health issues during their cancer trajectory, while social media recruitment may have skewed participation toward digitally active and health-seeking individuals. These factors may influence how participants interpret and discuss sexual health, potentially shaping the interview data. Additionally, the transferability of the results to young adults in other countries may be limited, as social, economic, and political contexts influence how sexual health is understood and experienced [63, 64]. A change of the present EORTC QLQ-SH22 questionnaire will depend on the enlarged and reperformed investigation of the relevance, comprehensibility and topics needing elaboration or missing when explored in a broader and larger international young adult patient cohort.

## Conclusions

This study highlights the relevance of the EORTC QLQ-SH22 to address sexual health in young adults with cancer while indicating the need for revisions to improve comprehensibility. Enhancements could include elaborating on topics such as intimacy, communication with professionals, body changes and body image, and partnership and incorporating missing topics like singlehood, cancer prior to sexual debut, contraception and fertility, and gender diversity. We recommend conducting a dedicated international content validity study within a young adult cancer population to guide future item development.

## Supplementary Information

Below is the link to the electronic supplementary material.

Supplementary Material 1

## Data Availability

The datasets used and/or analysed during the current study are available from the corresponding author on reasonable request.

## Abbreviations

AYA: Adolescent and young adult
ECOG: Eastern Cooperative Oncology Group
EORTC: European Organization for Research and Treatment of Cancer
EORTC: QLQ-C30 European Organisation for Research and Treatment of Cancer Quality of Life Questionnaire - Core 30
EORTC: QLQ-SH22 European Organisation for Research and Treatment of Cancer Quality of Life Questionnaire - Sexual Health 22
HCPs: Healthcare professionals
HR-QoL: Health-Related Quality of Life
PROM: Patient-Reported Outcomes Measurement
PROMIS: Patient-Reported Outcomes Measurement Information System
QoL: Quality of Life
SD: Standard Deviation
