# Supplementary material for: Exploration of the relevance and comprehensibility of the European Organization for the Research and Treatment of Cancer Sexual Health Questionnaire among Danish young adults aged 18–39: a national cross-sectional study
Source: J Patient Rep Outcomes. 2025 Dec 31;10:3. doi: 10.1186/s41687-025-00988-w (PMC12770101; doi:10.1186/s41687-025-00988-w)
Supplement: Supplementary file 1 — Supplementary Material 1 [file 41687_2025_988_MOESM1_ESM.docx]

**Supplementary file 1**

**Table S1: Grading of the relevance of each item in the EORTC QLQ-SH22**

| **Item** | **1: Not at all relevant**  **N (%)** | **2: A little relevant**  **N (%)** | **4: Quite a bit relevant**  **N (%)** | **4: Very relevant**  **N (%)** |
| --- | --- | --- | --- | --- |
| 1 How important to you is an active sex life? | 2 (3) | 4 (7) | 20 (35) | 34 (59) |
| 2 Have you had decreased libido? | 3 (5) | 2 (4) | 17 (3) | 38 (67) |
| 3 Have you been satisfied with your level of sexual desire? | 5 (8) | 10 (18) | 22 (40) | 23 (42) |
| 4 Have you been satisfied with your sex life? | 6 (10) | 14 (26) | 13 (24) | 27 (50) |
| 5 Have you been worried about being incontinent (urine/stool)? | 24 (40) | 8 (22) | 9 (25) | 19 (53) |
| 6 Has fatigue or a lack of energy affected your sex life? | 2 (3) | 3 (5) | 10 (17) | 45 (78) |
| 7 Has the treatment affected your sexual activity? | 5 (8) | 5 (9) | 11 (20) | 39 (71) |
| 8 Have you been worried that sex would be painful? | 8 (13) | 8 (15) | 13 (25) | 31 (60) |
| 9 Have you had communication with health professionals about sexual issues? | 7 (12) | 9 (17) | 19 (36) | 25 (47) |
| 10 Have you been satisfied with the communication about sexual issues between yourself and your partner? | 8 (13) | 8 (15) | 14 (27) | 30 (58) |
| 11 Have you been worried that your partner may cause you pain during sexual contact? | 10 (17) | 14 (28) | 15 (30) | 21 (42) |
| 12 Have you been satisfied with your level of intimacy? | 6 (10) | 7 (13) | 21 (39) | 26 (52) |
| 13 Have you felt insecure regarding your ability to satisfy your partner? | 3 (5) | 7 (12) | 12 (21) | 38 (67) |
| 14 For men only: Were you confident about obtaining and maintaining an erection when you had sex? | 0 (0) | 4 (11) | 9 (25) | 23 (64) |
| 15 For men only: Have you felt less masculine as a result of your disease or treatment? | 4 (11) | 5 (16) | 6 (19) | 21 (66) |
| 16 For women only: Have you felt less feminine as a result of your disease or treatment? | 0 (0) | 1 (4) | 3 (13) | 20 (83) |
| 17 During the last 4 weeks: Have you been sexually active? | 5 (8) | 4 (7) | 21 (38) | 30 (55) |
| 18 If yes in Q17: Has sexual activity been enjoyable for you? | 4 (7) | 4 (7) | 20 (36) | 32 (57) |
| 19 If yes in Q17: Have you been satisfied with your ability to reach an orgasm? | 5 (8) | 9 (16) | 18 (33) | 28 (51) |
| 20 If yes in Q17: Have you felt pain during/after sexual activity? | 10 (17) | 6 (12) | 15 (30) | 22 (44) |
| 21 If yes in Q17: To what extent did you feel sexual enjoyment? | 10 (17) | 10 (20) | 21 (42) | 19 (38) |
| 22 For women only: Have you experienced a dry vagina during sexual activity? | 2 (8) | 2 (9) | 6 (27) | 14 (64) |
